# SALMFamide2 and serotonin immunoreactivity in the nervous system of some acoels (Xenacoelomorpha)

**DOI:** 10.1002/jmor.20794

**Published:** 2018-02-01

**Authors:** Isabel L. Dittmann, Thomas Zauchner, Lucy M. Nevard, Maximilian J. Telford, Bernhard Egger

**Affiliations:** ^1^ Research unit Evolutionary Developmental Biology Institute of Zoology, University of Innsbruck, Technikerstr. 25 Innsbruck 6020 Austria; ^2^ Department of Genetics, Evolution and Environment University College London, Darwin Building, Gower Street London WC1E 6BT United Kingdom

**Keywords:** antibody specificity, immunocytochemistry, Nemertodermatida, nervous system, *Xenoturbella*

## Abstract

Acoel worms are simple, often microscopic animals with direct development, a multiciliated epidermis, a statocyst, and a digestive parenchyma instead of a gut epithelium. Morphological characters of acoels have been notoriously difficult to interpret due to their relative scarcity. The nervous system is one of the most accessible and widely used comparative features in acoels, which have a so‐called commissural brain without capsule and several major longitudinal neurite bundles. Here, we use the selective binding properties of a neuropeptide antibody raised in echinoderms (SALMFamide2, or S2), and a commercial antibody against serotonin (5‐HT) to provide additional characters of the acoel nervous system. We have prepared whole‐mount immunofluorescent stainings of three acoel species: *Symsagittifera psammophila* (Convolutidae), *Aphanostoma pisae*, and the model acoel *Isodiametra pulchra* (both Isodiametridae). The commissural brain of all three acoels is delimited anteriorly by the ventral anterior commissure, and posteriorly by the dorsal posterior commissure. The dorsal anterior commissure is situated between the ventral anterior commissure and the dorsal posterior commissure, while the statocyst lies between dorsal anterior and dorsal posterior commissure. S2 and serotonin do not co‐localise, and they follow similar patterns to each other within an animal. In particular, S2, but not 5‐HT, stains a prominent commissure posterior to the main (dorsal) posterior commissure. We have for the first time observed a closed posterior loop of the main neurite bundles in *S. psammophila* for both the amidergic and the serotonergic nervous system. In *I. pulchra*, the lateral neurite bundles also form a posterior loop in our serotonergic nervous system stainings.

## 1 INTRODUCTION

Acoels are small worm‐shaped animals, which can be mainly found in benthic marine habitats (Achatz & Martínez, 2012). Together with nemertodermatids, acoels constitute the taxon Acoelomorpha, originally part of the Platyhelminthes (Ehlers, 1985). Many molecular phylogenies have shown this phylogenetic position to be unlikely, with phylogenomic studies most often placing acoelomorphs—together with *Xenoturbella* as Xenacoelomorpha—either as sister group to Nephrozoa (Cannon et al., 2016; Hejnol et al., 2009; Rouse, Wilson, Carvajal, & Vrijenhoek, 2016; Srivastava, Mazza‐Curll, van Wolfswinkel, & Reddien, 2014) or within Deuterostomia as sister group to Ambulacraria (Philippe, Brinkmann, Martínez, Riutort, & Baguñà, 2007; Philippe et al., 2011).

The nervous system of xenacoelomorphs takes different shapes, ranging from a basiepidermal nerve layer without a distinct brain in *Xenoturbella* and some nemertodermatids, to a centralised nervous system with a so‐called commissural brain in some nemertodermatids and most acoels (Achatz & Martínez, 2012). In acoels, longitudinal neurite bundles stretch from the brain to the posterior, and a peripheral, submuscular, serotonergic plexus extends throughout the animals. The commissural brain shows substantial differences between acoel groups, but it takes one of three main shapes: barrel, rosette, or bridge (Reuter, Raikova, Jondelius, et al., 2001). The number of commissures constituting the brain and the number of longitudinal neurite bundles is a character of use for systematics (Achatz & Martínez, 2012). In most acoels studied so far, three pairs of longitudinal neurite bundles are present, but this number can range from 2 to 6 neurite bundles (Raikova, Reuter, Kotikova, & Gustafsson, 1998). Commissures posterior to the brain are not distinct and are usually restricted to the peripheral plexus (Gaerber, Salvenmoser, Rieger, & Gschwentner, 2007; Raikova et al., 1998).

In acoels, the serotonergic nervous system has been studied most extensively, using antibodies against 5‐hydroxytryptamine (5‐HT). The majority of studies deals with relatively few genera: several species of the genera *Childia* and *Convolutriloba*, and eight species of other genera (see Table 1). Another fairly well explored part of the acoels’ nervous system concerns the RFamidergic nervous system, using antibodies against the mollusc FMRFamide, the flatworm GYIRFamide and a simple RFamide (see Table 1).

**Table 1:** Overview of papers dealing with 5‐HT and RFamide immunocytochemistry (ICC) in acoels. Modified after Haszprunar (2016)

ICC	Species	Authors
5‐HT	*Actinoposthia beklemischevi*	Kotikova and Raikova (2008); Raikova et al. (1998)
	*Anaperus biaculeatus*	Raikova et al. (1998)
	*Aphanostoma pisae*	Zauchner et al. (2015)
	*Avagina incola*	Reuter, Raikova, and Gustafsson (2001); Reuter, Raikova, Jondelius, et al. (2001)
	*Childia brachyposthium*	Raikova et al. (2004a)
	*Childia crassum*	Reuter, Raikova, and Gustafsson (2001); Reuter, Raikova, Jondelius, et al. (2001); Raikova et al. (2004a)
	*Childia cycloposthium*	Raikova et al. (2004a)
	*Childia groenlandica*	Raikova et al. (1998); Reuter, Raikova, and Gustafsson (2001)
	*Childia macroposthium*	Raikova et al. (2004a)
	*Childia submaculatum*	Raikova et al. (2004a)
	*Childia trianguliferum*	Raikova et al. (2004a)
	*Convolutriloba hastifera*	Sikes and Bely (2008)
	*Convolutriloba longifissura*	Gaerber er al. (2007); Hejnol and Martindale (2008); Sikes and Bely (2008);
	*Convolutriloba macropyga*	Sikes and Bely (2008)
	*Convolutriloba retrogemma*	Sikes and Bely (2008, 2010)
	*Faerlea glomerata*	Reuter, Raikova, and Gustafsson (2001); Reuter, Raikova, Jondelius, et al. (2001)
	*Isodiametra pulchra*	Achatz and Martínez (2012); Achatz, Chiodin, Salvenmoser, Tyler, and Martínez (2013); Moreno, De Mulder, Salvenmoser, Laduner, and Martínez (2010)
	*Mecynostomum* sp.	Raikova et al. (1998)
	*Symsagittifera roscoffensis*	Bailly et al. (2014); Bery, Cordona, Martínez, and Hartenstein (2010); Semmler et al. (2010)
FMRFamide	*Actinoposthia beklemischevi*	Kotikova and Raikova (2008); Reuter, Raikova, and Gustafsson (1998)
	*Anaperus biaculeatus*	Reuter et al. (1998)
	*Avagina incola*	Reuter, Raikova, and Gustafssonl (2001)
	*Childia groenlandica*	Reuter et al. (1998)
	*Childia crassum*	Reuter, Raikova, and Gustafsson (2001)
	*Mecynostomum* sp.	Reuter et al. (1998)
	*Isodiametra pulchra*	Achatz and Martínez (2012)
GYIRFamide	*Avagina incola*	Reuter, Raikova, Jondelius, et al. (2001)
	*Childia brachyposthium*	Raikova et al. (2004a)
	*Childia crassum*	Raikova et al. (2004a); Reuter, Raikova, Jondelius, et al. (2001)
	*Childia macroposthium*	Raikova et al. (2004a)
Rfamide	*Symsagittifera roscoffensis*	Lechauve et al. (2013); Semmler et al. (2010)

The neuropeptide S2 is a dodecapeptide with the amino acid sequence Ser‐Gly‐Pro‐Tyr‐Ser‐Phe‐Asn‐Ser‐Gly‐Leu‐Thr‐Phe‐NH_2_ (SGPYSFNSGLTFamide), first purified from two starfish species, *Asterias rubens* and *Asterias forbesi* (Elphick, Price, Lee, & Thorndyke, 1991). Besides several echinoderms, the S2 antibody has been shown to stain parts of the nervous system of *Xenoturbella bocki* and the enteropneust *Harrimania kupfferi*, and it has been interpreted as supporting a deuterostome affiliation of *Xenoturbella* (Stach et al., 2005).

The posterior part of the acoel nervous system has frequently been found to be particularly difficult to visualise (Gaerber et al., 2007; Semmler, Chiodin, Bailly, Martínez, & Wanninger, 2010; Zauchner, Salvenmoser, & Egger, 2015). Here, we test 5‐HT and S2 immunoreactivity in several acoel species to explore new details of the acoel nervous system, and to determine whether positive S2 immunoreactivity is present in other members of Xenacoelomorpha besides *Xenoturbella*.

## 2 MATERIALS AND METHODS

### 2.1 Animals

*Macrostomum lignano*, Ladurner, Schärer, Salvenmoser, and Rieger, 2005, *Isodiametra pulchra* (Smith and Bush, 1991) and *Aphanostoma pisae* (Zauchner, Salvenmoser, & Egger, 2015) are maintained in permanent lab cultures and are fed with the diatom *Nitzschia curvilineata* Hustedt, 1922 (Egger & Ishida, 2005; Zauchner et al., 2015). *Symsagittifera psammophila* (Beklemischev, 1957) was extracted from sand samples from Marina di Pisa, Italy, and undetermined juvenile bivalves were extracted from marine samples taken in Punat and Rovinj, Croatia. *Maritigrella crozieri* (Hyman, 1939) and its larvae were collected and cultured as described by Lapraz et al. (2013).

### 2.2 Immunocytochemistry

Live animals were relaxed for 5–10 min in 7.14% MgCl_2_*6H_2_O and then fixed at room temperature in 4% formaldehyde in PBS for 1 hr. The whole‐mounts were washed in phosphate buffered saline (PBS) with 0.1% Triton‐X100 (PBS‐T) for 30 min with multiple changes of solutions. The specimens were blocked for 3 hr in PBS‐T with 1% bovine serum albumin (BSA‐T) before being incubated in primary antibody diluted in BSA‐T at 4°C overnight. Several dilutions of the monoclonal primary antibody against S2 (raised in rabbit) (Newman, Elphick, & Thorndyke, 1995) have been tested (1:100; 1:500; 1:800, 1:1,500; 1:1,600,1:2,000; 1:3,200, 1:10,000; 1:20,000), the best results were achieved with a dilution of 1:500. In *M. lignano* and *M. crozieri*, dilutions of 1:800, 1:1,600, and 1:3,200, and in the juvenile bivalves a dilution of 1:500 were tested. The third use of the recycled antibody dilution resulted in the best staining. The primary antibody against 5‐HT (monoclonal mouse anti‐human serotonin clone 5HT‐H209 DakoCytomation) was used in a dilution of 1:25 in BSA‐T for *I. pulchra* and *S. psammophila*. For double stainings, both primary antibodies were applied simultaneously. Afterwards, whole‐mounts were washed in PBS‐T overnight at 4°C and blocked in BSA‐T for 2 hr at room temperature and incubated in a 1:300 dilution of the secondary antibody (Alexa Fluor 488 goat anti‐rabbit or Alexa Fluor 555 goat anti‐mouse) for 1 hr at room temperature. Specimens were washed again for at least 30 min in PBS‐T, best results were achieved by washing over three nights at 4°C. For observing the samples under the microscope, whole‐mounts were mounted in VectaShield (Vector Laboratories). The primary antibody was omitted in negative controls.

### 2.3 Documentation

Specimens were photographed with a Leica DM 5000B compound microscope equipped with a Leica DFC 490 digital camera and with a Leica TCS SP5 IIT confocal laser scanning microscope. Further image processing was performed with Fiji and Adobe Photoshop CS2. Drawings were produced in Adobe Illustrator CS2. Stainings are shown in false colours to accommodate colour‐blindness.

## 3 RESULTS

### 3.1 Immunoreactivity to an S2 antibody

**Figure 1:**
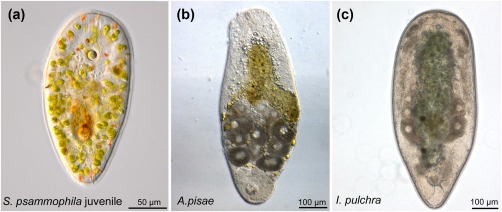
Live images of the three acoel species used in this study: (a) *Symsagittifera psammophila*, (b) *Aphanostoma pisae*, (c) *Isodiametra pulchra*. Note that in (a) a juvenile is shown, while stainings have been performed with adults in all cases

**Figure 2:**
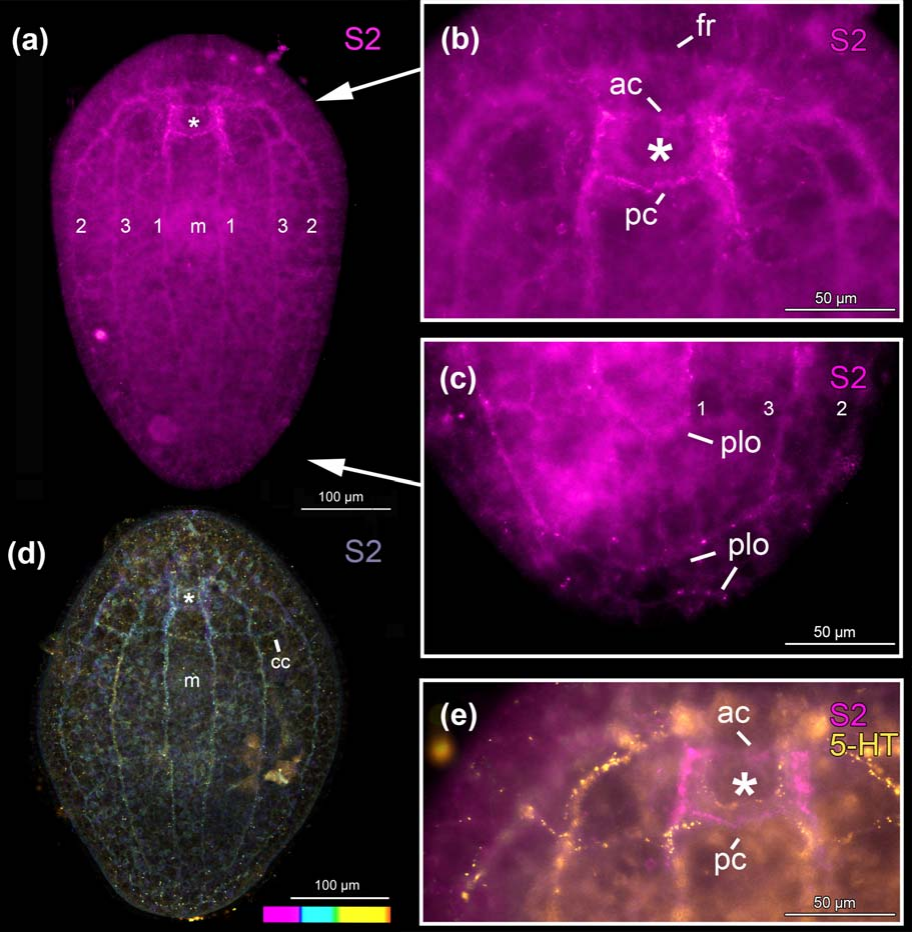
*Symsagittifera psammophila* stained with S2 antibody (a–e, magenta) and 5‐HT antibody (e, yellow). (a) Projection of whole‐mount. (b) Projection of the anterior body‐part. (c) Projection of the posterior body‐part. (d) Confocal projection with temporal colour coding; colour‐code: pink (dorsal) to orange/red (ventral). (e) Overlay projection of the anterior body‐part of a double‐labelled animal. (a–c, e) Same individual. Abbreviations: 1 dorsal neurite bundle; 2 lateral neurite bundle; 3 ventral neurite bundle; ac dorsal anterior commissure; cc collum commissure; fr frontal ring; m mouth; pc dorsal posterior commissure; plo posterior loop. Asterisk marks position of statocyst

**Figure 3:**
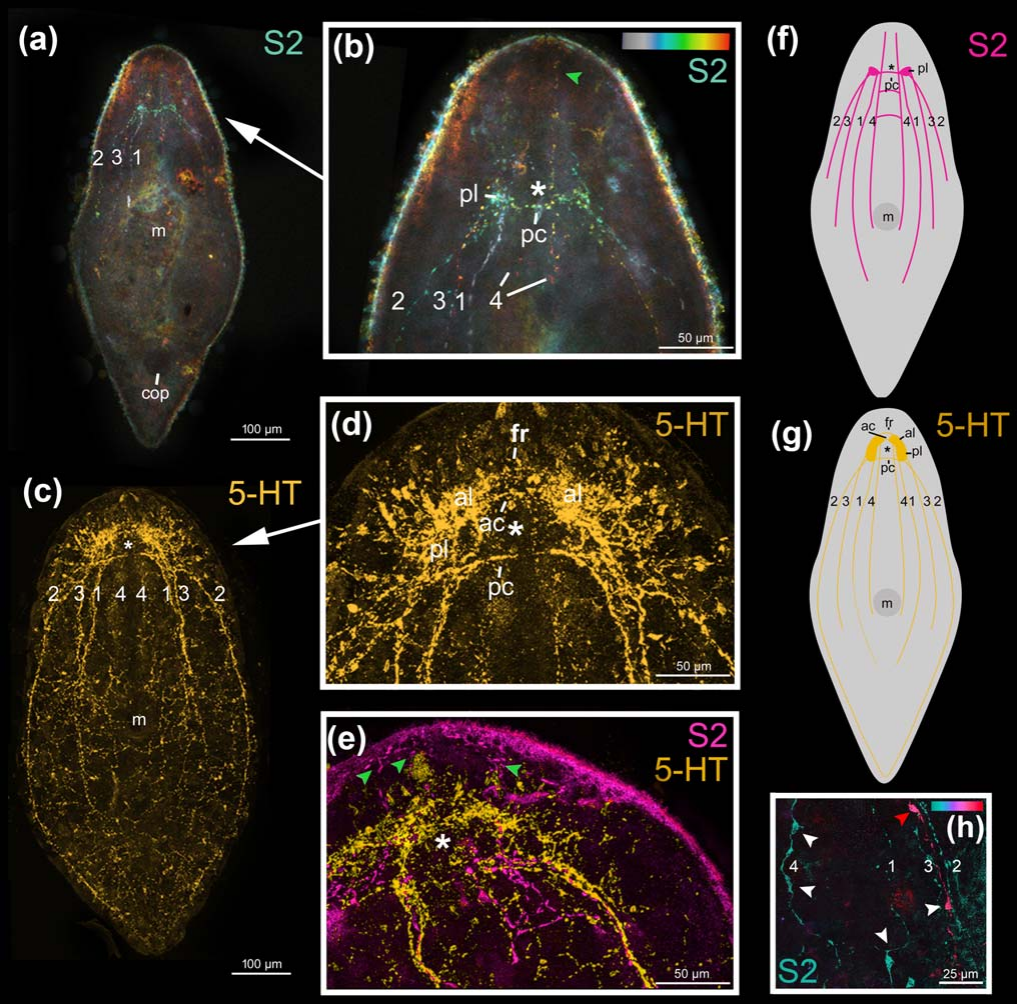
Confocal projection of *Isodiametra pulchra* stained with S2 antibody (a,b,e, magenta) and 5‐HT (c–e, yellow). (a) Confocal projections of a whole‐mount and (b) of the anterior body part with temportal colour coding, colour‐code: grey (dorsal) to red (ventral). Green arrowhead points to delicate anterior process of neurites. (c) Confocal projections of a whole‐mount and (d) of the anterior body‐part. (e) Overlay projection of the anterior body part of a double‐labelled animal. Green arrowheads point to delicate anterior process of neurites. (f‐g) Schematic drawings of the nervous system stained with S2 (f) and 5‐HT (g) antibodies. (h) Detailed view of bipolar and multipolar (red and white arrowheads, respectively) neurons of the colour‐coded (blue dorsal, red ventral) S2amidergic nervous system. (a,b), (c,d), (e,h) same individuals. Abbreviations: 1 dorsal neurite bundle; 2 lateral neurite bundle; 3 ventral neurite bundle; 4 medio‐ventral neurite bundle; ac: dorsal anterior commissure; al anterior lobe; cop male copulatory organ; fr frontal ring; m mouth; pc dorsal posterior commissure; pl posterior lobe. Asterisks mark position of statocyst

**Figure 4:**
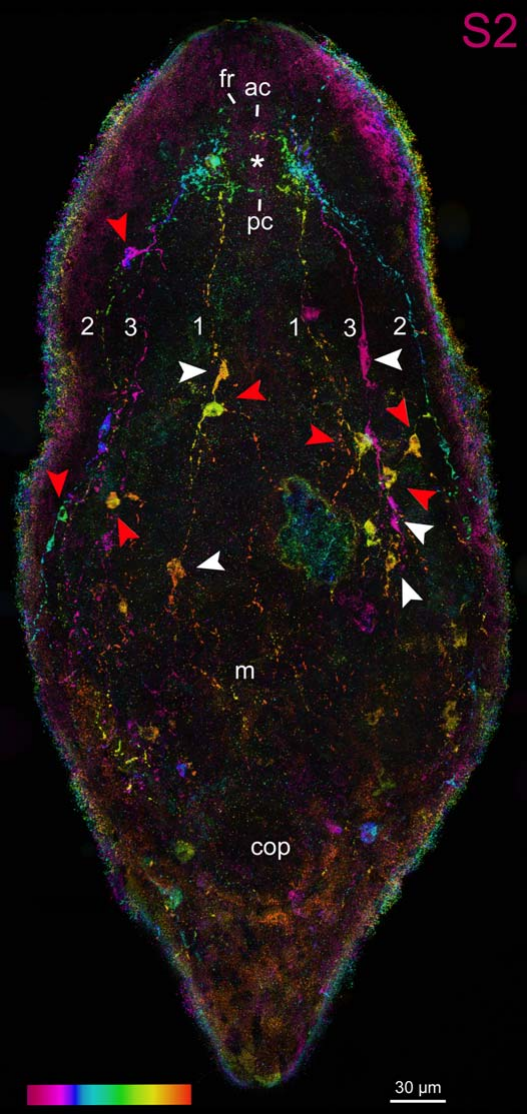
Confocal projection of a whole‐mount *Aphanostoma pisae* stained with S2 antibody with temporal colour coding. Colour‐code: pink (ventral) to red (dorsal), arrowheads point to putative neuronal cell bodies of bipolar (white) or multipolar (red) neurons. Abbreviations: 1 dorsal neurite bundle; 2 lateral neurite bundle; 3 ventral neurite bundle; ac dorsal anterior commissure; cop male copulatory organ; fr frontal ring; m mouth; pc dorsal posterior commissure. Asterisk marks the position of the statocyst

**Figure 5:**
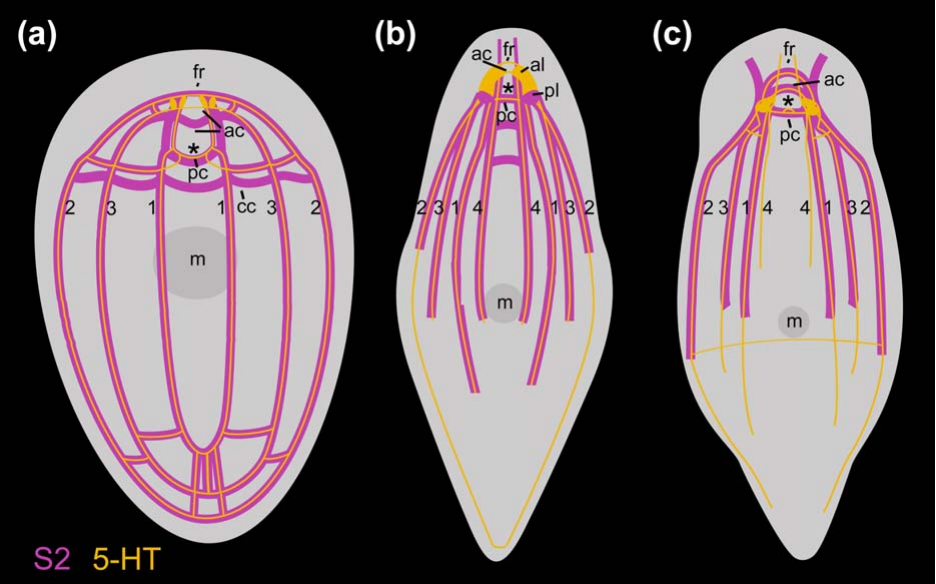
Schematic drawings of the nervous systems stained with S2 (magenta) and 5‐HT (yellow) antibodies. (a) *Symsagittifera psammophila*. (b) *Isodiametra pulchra*. (c) *Aphanostoma pisae* 5‐HT (yellow) after Zauchner et al. (2015). Abbreviations: 1 dorsal neurite bundle; 2 lateral neurite bundle; 3 ventral neurite bundle; 4 medio‐ventral neurite bundle; ac dorsal anterior commissure; al anterior lobe; cc collum commissure; fr frontal ring; pc dorsal posterior commissure; pl posterior lobe. Asterisks mark position of statocyst

In *Symsagittifera psammophila, Isodiametra pulchra*, *and Aphanostoma pisae* (Figure 1), the nervous system shows positive immunoreactivity with the antibody raised against S2 (Figures 2, 3a,b,e,f, 4, and 5). S2 immunoreactivity can be observed in the commissural brain of all three species. In *I. pulchra*, only the dorsal posterior commissure of the commissural brain and the posterior, but not the anterior lobe shows immunoreactivity to S2 (Figure 3B). This is in contrast to *S. psammophila* and *A. pisae* which have two labelled commissures, between which the statocyst is located (Figures 2b and 4). Both in *A. pisae* and *S. psammophila*, but not in *I. pulchra*, the frontal ring (comprised of ventral anterior commissure and dorsal anterior commissure, see Sprecher et al., 2015) is labelled (Figures 2, 4, and 5). The dorsal, the lateral, as well as the ventral neurite bundles can be observed in all three species (Figures 2a, 3b, and 4). In *I. pulchra*, the medio‐ventral neurite bundle is stained as well and delicate anterior processes can be observed (Figure 3b). We observe bipolar neurons in the dorsal, ventral and medio‐ventral neurite bundles of *I. pulchra* (Figure 3h), and in the dorsal and ventral neurite bundles of *A. pisae* (Figure 4). Multipolar neurons with three branches are detected in the ventral neurite bundles of *I. pulchra* (Figure 3h) and in all neurite bundles of *A. pisae* (Figure 4). In *S. pisae* and in the brain of *I. pulchra* and *A. pisae*, the individual neurons are not clearly identifiable. Branch lengths of neurons are measured between about 8 and 60 µm in *I. pulchra* and between 8 and 80 µm in *A. pisae*. In the posterior of *S. psammophila* the dorsal, lateral and ventral neurite bundles each form a loop, interconnected by several short longitudinal processes (Figures 2a,c,d). Posterior to the dorsal posterior commissure, another commissure, stretching continuously from one lateral neurite bundle to the other, is apparent in *S. psammophila* (Figure 2d). Between the median neurite bundles, the commissure slightly sinks to the ventral side. We termed this commissure the collum commissure. A subepidermal nerve net could not be observed in any of the three acoels. The S2amidergic nervous system is schematised in Figures 3f and 5.

*Macrostomum lignano*, larvae of *Maritigrella crozieri* (both Platyhelminthes), juvenile molluscs, and negative controls on acoels did not show any specific staining.

### 3.2 Immunoreactivity to serotonin

**Figure 6:**
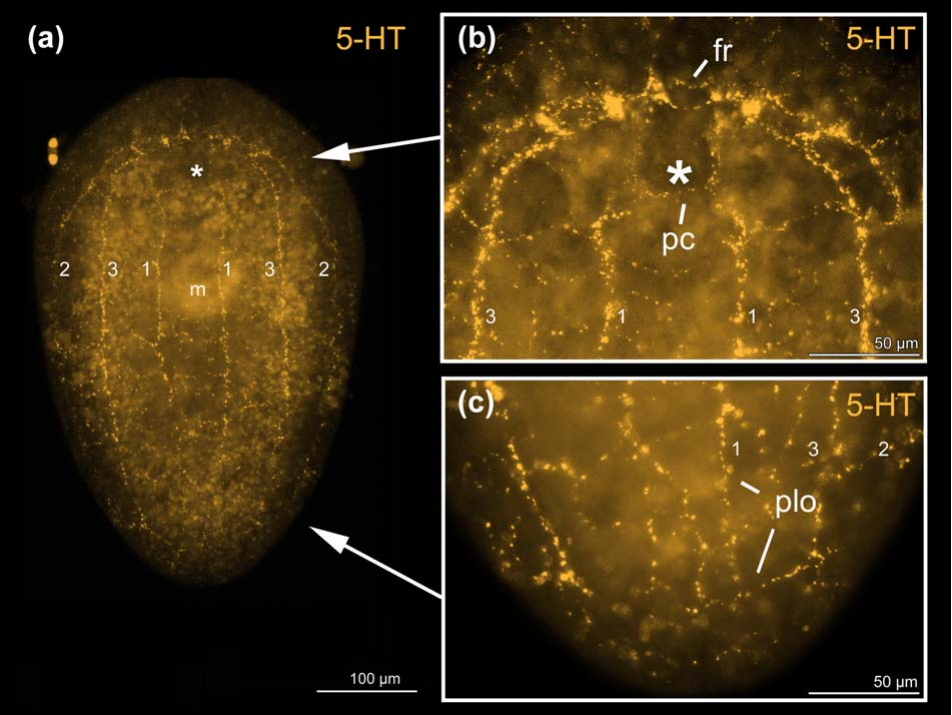
*Symsagittifera psammophila* stained with 5‐HT antibody. Projections of a whole‐mount (a), of the anterior body‐part (b) and of the posterior body‐part (c). (a–c) Same individual. Abbreviations: 1 dorsal neurite bundle; 2 lateral neurite bundle; 3 ventral neurite bundle; ac dorsal anterior commissure; fr frontal ring; pc dorsal posterior commissure; plo posterior loop. Asterisks mark position of statocyst

Both acoel species, *S. psammophila* and *I. pulchra*, exhibit positive immunoreactivity to serotonin (Figures 3c–e,g, 5 and 6). A peripheral plexus permeates the animals with very narrow processes, which are concentrated around the mouth (Figures 3c and 6a). The commissural brain comprises a frontal ring and an anterior and a dorsal posterior commissure, which are immunoreactive in the two species, but there are some morphological differences concerning the anterior and posterior lobes. In *I. pulchra*, both anterior and posterior lobes are labelled (Figure 3c–e,g), while in *S. psammophila* only the anterior lobes are visible, with an anterolateral lobe on either side (Figures 2e and 6b). The dorsal and the lateral neurite bundles as well as the ventral neurite bundle can be observed in both species (Figures 3c,g, 5a, and 6a,b). Only in *I. pulchra*, the medio‐ventral neurite bundles can be observed (Figures 3c,g, 5b). In the posterior body‐part of *S. psammophila* the dorsal, lateral and ventral neurite bundles each form a loop, interconnected by several short longitudinal processes (Figures 5a and 6a,c). The dorsal neurite bundles close the loop most anteriorly, followed by the ventral neurite bundles and finally the lateral neurite bundles. Similarly, in *I. pulchra* the lateral neurite bundles form a loop close to the posterior tip of the animal (Figure 3c,g). The dorsal posterior commissure spans between the lateral neurite bundles, but no commissures more posterior to the dorsal posterior commissure were detected.

The serotonergic nervous system of *S. psammophila* and *I. pulchra* is very similar to the respective S2 stainings of these species (Figures 2e, 3e, and 5a,b), with the following exceptions:

- In both species, the peripheral nerve plexus is only stained by an antibody against serotonin, but not by an antibody against S2.
- In *I. pulchra* serotonergic staining can also be observed in the frontal ring, the anterior lobe and the dorsal anterior commissure which are parts of the commissural brain (Figures 3d and 5b). Also, the posterior loop of the lateral neurite bundles can only be seen with serotonin.
- In the posterior body‐part of *S. psammophila*, the three loops of the main neurite bundles and their interconnections are less pronounced in the serotonergic immunoreactivity (Figure 6c). The collum commissure is specific to the S2 immunoreactivity and missing in the serotonergic nervous system (Figures 2d, 5a, and 6a). Finally, the serotonergic pattern around the statocyst is weaker than the S2 immunoreactivity (Figures 2e and 5a) in *S. psammophila*.
- Our serotonergic nervous system stainings do not readily reveal individual neurons and the number of their processes, while this is possible in S2 stainings of *I. pulchra* (but not of *S. psammophila*).

Negative controls omitting the primary antibody did not show any staining. A comparative overview of both S2 and serotonin stainings in all three acoel species is provided (Figures 3f,g and 5).

## 4 DISCUSSION

### 4.1 Comparison of nervous system patterns

The present study documents the staining of parts of the nervous system in the three acoel species *Symsagittifera psammophila, Isodiametra pulchra*, and *Aphanostoma pisae* using a monoclonal antibody raised against the echinoderm neuropeptide S2, with the specific peptide sequence used for antibody production being KYSGLTFamide (Newman et al., 1995). Additionally, we have stained the serotonergic (5‐HT) nervous system in *S. psammophila* and *I. pulchra*. No staining of the serotonergic nervous system of *A. pisae* was performed as this has been recently published (Zauchner et al., 2015). In *A. pisae*, the main differences between the S2amidergic and the serotonergic pattern of the nervous system lie in the position of the anterior processes, where the serotonergic processes are situated towards the midline, and the S2amidergic processes are situated more laterally (Zauchner et al., 2015; Figures 4 and 5c). Also, the peripheral nerve plexus typical for serotonin is missing in the S2 stainings. Even if the stainings of the nervous system with antibodies raised against S2 and serotonin show a similar pattern, they are not identical and do not co‐localise either in *S. psammophila* or in *I. pulchra* (Figures 2e and 3e). This is especially apparent in the dorsal anterior commissure of *S. psammophila*, which is located more anteriorly in the serotonergic pattern than in the S2 stainings (Figure 5). Also, individual neurons could only be identified in S2 stainings of *I. pulchra* and *A. pisae*, but not in the respective 5‐HT stainings, whereas in *S. psammophila*, neither S2 nor 5‐HT stainings revealed individual neurons. Possibly, the smaller size of the isodiametrid species allows for easier analysis of the nervous system. FMRFamide and 5‐HT were shown to not co‐localise in *I. pulchra* (Achatz & Martínez, 2012), and in other acoels, GYIRFamide and 5‐HT stainings were also found to be similar, but not to co‐localise (Raikova et al., 2004a). We have identified bipolar and multipolar neurons in the S2‐stained neurite bundles of both, *I. pulchra* and *A. pisae*. This agrees with findings in *Xenoturbella bocki*, where cells expressing FMRFamide and SALMFamide are bipolar or multipolar (Martínez et al., 2017). In *I. pulchra*, the serotonergic nervous system shown here (Figure 3c,d,g) is in concordance with the description of Achatz and Martínez (2012), except for the posterior loop we found in our stainings. Different to the serotonergic, but similar to the synaptotagminergic nervous system of adult *S. roscoffensis* (Gavilán, Perea‐Atienza, & Martínez, 2016; Semmler et al., 2010), in *S. psammophila* recognisable posterior loops of the longitudinal serotonergic neurite bundles are visible (Figures 4a and 5a,c), which are also evident in the respective S2 stainings (Figures 2a,c,d and 5a).

In sea stars, S2 was found to be an important agent to relax the cardiac stomach (Elphick et al., 1991; Newman et al., 1995). While no functional studies in acoels have been performed to date, the overall distribution of the nervous system stained with the S2 antibody suggests a broader role of an S2‐like neuropeptide in acoels.

### 4.2 Posterior loops and commissures in acoels

In *Symsagittifera psammophila*, all three pairs of main neurite bundles form a posterior loop, while in *Isodiametra pulchra*, only the lateral neurite bundles join in a posterior loop (Figure 5). So far, posterior loops of the main neurite bundles as shown in *S. psammophila* and *I. pulchra* have not been described as such for either the genus *Symsagittifera* (Semmler et al., 2010), or in *I. pulchra*, or in fact, for any other acoel species. However, a recent publication using an antibody against synaptotagmin in *Symsagittifera roscoffensis* shows and describes that the three pairs of neurite bundles ‘converge at the posterior end’ (Gavilán et al., 2016). In two other acoels, *Anaperus biaculeatus* and *Actinoposthia beklemischevi*, the acetylcholinergic nervous system also reveals posterior loops of the (ventro)lateral and the dorsolateral neurite bundles (Raikova et al., 1998). It may be that posterior loops in other acoels are present, but possibly remain unrecognised, as often the staining of the nervous system in acoels gets substantially weaker towards the posterior end (Zauchner et al., 2015). This observation coincides with the expression pattern of the transcription factor and neural marker gene *SoxB1* in *S. roscoffensis*, which is predominantly expressed in the anterior body half of juveniles, reflecting the concentration of the nervous system in this region (Semmler et al., 2010).

A closed posterior loop of the main neurite bundles is a common feature in eumetazoans and has also been described for the nemertodermatid *Meara stichopi* (Raikova, Reuter, Jondelius, & Gustafsson, 2000), whereas in another nemertodermatid, *Nemertoderma westbladi*, such a loop is not apparent (Raikova et al., 2004b; Raikova, Meyer‐Wachsmuth, & Jondelius, 2016). In *Xenoturbella*, only a basiepidermal nerve plexus, but no neurite bundles are present (Gavilán et al., 2016).

We have identified a commissure posterior of the brain, spanning between the lateral neurite bundles in *S. psammophila*, which we termed the collum commissure (Figures 2d and 5a). This commissure could only be detected in the S2amidergic, but not in the serotonergic nervous system. In *S. roscoffensis*, a similar (unnamed) commissure was found, disjointedly connecting the main neurite bundles, both in the serotonergic and synaptotagminergic nervous systems (Gavilán et al., 2016; Semmler et al., 2010). Apart from the commissural brain, commissures between the lateral neurite bundles are rare in acoels and have been detected in *Aphanostoma pisae*, but not in the closely related *Isodiametra pulchra* (Zauchner et al., 2015). In the nemertodermatid *Meara stichopi*, such commissures occur at regular intervals in the serotonergic nervous system, especially in the anterior half of the body (Raikova et al., 2000). The size of the animals does not seem to play a role in the absence or presence of these commissures, as they can be found both in rather large (*Symsagittifera*, several mm in length) and small (*A. pisae*, less than a mm long) species.

### 4.3 Homology of the nervous system of the three observed acoel species

All three acoel species used here belong to the large taxon Crucimusculata, encompassing all acoels except Solenofilomorphidae, Hofsteniidae, Paratomellidae and Diopisthoporidae (Jondelius, Wallberg, Hooge, & Raikova, 2011). While both, *Isodiametra pulchra* and *Aphanostoma pisae*, are members of the same family, Isodiametridae, *Symsagittifera psammophila* belongs to another large and well‐studied family, the Convolutidae (Jondelius et al., 2011). This implies that the nervous system of *I. pulchra* and *A. pisae* are likely to be more similar to each other, than to *S. psammophila*.

We have found some general patterns in isodiametrid and in convolutid (serotonergic) nervous system architectures. In most studied isodiametrids, four pairs of longitudinal neurite bundles [exception: *Avagina incola* with two (Reuter, Raikova, Jondelius, et al., 2001)] are reported, from inner to outer bundle: (medio)ventral, dorsal, ventral/ventrolateral, and lateral (*Faerlea glomerata* in Reuter, Raikova, Jondelius, et al., 2001; *I. pulchra* in Achatz & Martínez, 2012 and this study; *A. pisae* in Zauchner et al., 2015). Interestingly, in our S2 stainings of *A. pisae*, we have only detected three pairs of longitudinal neurite bundles (Figure 4).

Three pairs of longitudinal neurite bundles are reported in *Convolutriloba longifissura* (dorsal, dorsal, median in Gaerber et al., 2007), *Symsagittifera roscoffensis* (median, lateral, lateral in Semmler et al., 2010), and in *Symsagittifera psammophila* (dorsal, ventral, lateral, this study). Given these species, there seems to be a higher variability in the location of the neurite bundles along the dorso‐ventral axis, as the second neurite bundle in *C. longifissura* is described as being ventral, in *S. psammophila* it is dorsal.

It is unclear which longitudinal neurite bundles in Isodiametridae are homologous to the ones found in Convolutidae and given the variability in the Convolutidae, the position along the dorso‐ventral axis is probably not a good indicator of homology.

According to the seminal study of Achatz and Martínez (2012), the dorsal posterior commissure behind the statocyst and the frontal ring anterior to the statocyst are likely homologous between members of the Crucimusculata. The frontal ring is also apparent in *Aphanostoma pisae* (Figure 5), although it was not named as such in the description of the serotonergic nervous system (Zauchner et al., 2015).

### 4.4 Specificity of the S2 antibody

In all three acoel species, parts of the nervous system can be stained by an antibody raised against echinoderm S2. This antibody has been demonstrated to work in a variety of echinoderms, a hemichordate and *Xenoturbella bocki*, but not in chordates, lophotrochozoans, crustaceans, and cnidarians (Stach et al., 2005). This is also corroborated by our own failed staining attempts in two flatworm species and in juvenile bivalves. With more studied species and groups, it will become clearer if positive immunoreactivity to the S2 antibody is truly restricted to ambulacrarians and xenacoelomorphs. For example, the neuropeptides SSLFFamide in the mollusc *Lottia gigantea* and SGLFFamide in the annelids *Capitella teleta* and *Helobdella robusta* match the *Asterias* SALMFamide pattern ‘S?[LF]?FG’ (Veenstra, 2011) and are prime candidates for possible affinity to the S2 antibody, even though two other annelids and a mollusc did not show positive S2 immunoreactivity (Stach et al., 2005 and our own findings).

## AUTHOR CONTRIBUTIONS

BE and MJT designed the study. ID, LN, TZ and BE performed antibody stainings and analysed the data. ID, BE and MJT wrote the manuscript. All authors agreed on the final manuscript.

## CONFLICT OF INTERESTS

The authors declare that no competing financial interests exist.
